# Exploring the heterogeneity of osteosarcoma cell characteristics and metabolic states and their association with clinical prognosis

**DOI:** 10.3389/fimmu.2024.1507476

**Published:** 2024-12-06

**Authors:** Sen Qin, YaoFeng Hu, RuCui Deng, Zhe Wang

**Affiliations:** ^1^ Department of Orthopedics, The First Affiliated Hospital of YangTze University, Jingzhou, Hubei, China; ^2^ Department of Neurological Care Unit, The First Affiliated Hospital of YangTze University, Jingzhou, Hubei, China

**Keywords:** osteosarcoma, metabolic pathways, comprehensive analysis, immune infiltration, prognostic analysis

## Abstract

**Background:**

Osteosarcoma is a malignant tumor originating from mesenchymal bone tissue, characterized by high malignancy and poor prognosis. Despite progress in comprehensive treatment approaches, the five-year survival rate remains largely unchanged, highlighting the need to clarify its underlying mechanisms and discover new therapeutic targets.

**Methods:**

This study utilized RNA sequencing data from multiple public databases, encompassing osteosarcoma samples and healthy controls, along with single-cell RNA sequencing data. Various methods were utilized, such as differential expression analysis of genes, analysis of metabolic pathways, and weighted gene co-expression network analysis (WGCNA), to pinpoint crucial genes. Using this list of genes, we developed and validated a prognostic model that incorporated risk signatures, and we evaluated the effectiveness of the model through survival analysis, immune cell infiltration examination, and drug sensitivity evaluation.

**Results:**

We analyzed gene expression and metabolic pathways in nine samples using single-cell sequencing data. Initially, we performed quality control and clustering, identifying 21 statistically significant cell subpopulations. Metabolic analyses of these subpopulations revealed heterogeneous activation of metabolic pathways. Focusing on the osteoblastic cell subpopulation, we further subdivided it into six groups and examined their gene expression and differentiation capabilities. Differential expression and enrichment analyses indicated that tumor tissues were enriched in cytoskeletal and structural pathways. Through WGCNA, we identified core genes negatively correlated with four highly activated metabolic pathways. Using osteosarcoma patient data, we developed a risk signature model that demonstrated robust prognostic predictions across three independent cohorts. Ultimately, we performed a thorough examination of the model, which encompassed clinical and pathological characteristics, enrichment analysis, pathways associated with cancer markers, and scores of immune infiltration, highlighting notable and complex disparities between high-risk and low-risk populations.

**Conclusion:**

This research clarifies the molecular mechanisms and metabolic features associated with osteosarcoma and how they relate to patient outcomes, offering novel perspectives and approaches for targeted therapy and prognostic assessment in osteosarcoma.

## 1 Introduction

Osteosarcoma, also known as osteogenic sarcoma, is a malignant tumor originating from mesenchymal bone tissue, characterized by its ability to produce bone-like tissue or bone matrix (1, 2). Osteosarcoma is the most prevalent primary malignant bone cancer, particularly affecting adolescents with a notable frequency (3, 4). This condition demonstrates a considerable level of aggressiveness and frequently results in an unfavorable prognosis, creating a considerable strain on both patients and their families. The treatment of osteosarcoma is constrained by tumor characteristics, chemosensitivity, and the challenges posed by post-recurrence therapy. Despite recent advances in comprehensive treatment modalities, including surgery, chemotherapy, and radiotherapy, the five-year survival rate for osteosarcoma patients has not seen significant improvement, leaving them facing substantial therapeutic challenges (5). Therefore, exploring the pathogenesis of osteosarcoma and identifying new therapeutic targets is of utmost importance (6, 7).

Carbohydrate metabolism, lipid metabolism, and nucleotide metabolism are critical for maintaining normal biological functions in cells, including tumor cells (8). The biosynthesis of glycosylphosphatidylinositol (GPI) anchors represents a significant post-translational modification that facilitates the attachment of non-transmembrane proteins to the outer layer of the plasma membrane (9). This modification plays a role in various biological functions, including signal transduction, cell adhesion, transport, and metabolism (10). On the other hand, the synthesis of glycosaminoglycans, especially heparan sulfate/heparin, is a multifaceted process characterized by the enzymatic action of several different enzymes (11). Glycosaminoglycans consist of linear polysaccharide chains made up of repeating disaccharide units (12). Heparan sulfate/heparin is critical for providing both structural support and regulatory functions within the extracellular matrix and on cell surfaces, influencing a wide array of biological activities, such as adhesion between cells, signaling processes, blood clotting, and the formation of new blood vessels. Glycolysis and gluconeogenesis are core pathways in the body’s energy metabolism. Glycolysis is the process of breaking down glucose into pyruvate, generating ATP, while gluconeogenesis refers to the conversion of non-carbohydrate precursors into glucose or glycogen (13, 14). Both glycolysis and gluconeogenesis play crucial roles in maintaining blood glucose levels, providing energy, and regulating metabolic balance. The biosynthesis of unsaturated fatty acids also involves the catalytic activity of various enzymes (15). Unsaturated fatty acids are important components of cell membranes, critical for maintaining membrane fluidity and stability (16, 17). Furthermore, unsaturated fatty acids are involved in various biological processes such as signal transduction, cell adhesion, and inflammatory responses. Given that osteosarcoma is a malignant connective tissue tumor, its development may be associated with disturbances in the aforementioned metabolic and biosynthetic processes.

To enhance our comprehension of the regulatory mechanisms governing gene expression in osteosarcoma and its association with cellular metabolism, we acquired bulk RNA sequencing data from both osteosarcoma tissues and healthy control samples sourced from various public databases (18). With this data, we performed differential expression analysis, enrichment analysis, and weighted gene co-expression network analysis (WGCNA) to identify genes with aberrant expression, relevant pathways, and gene co-expression modules linked to osteosarcoma (19). This foundational work will facilitate subsequent functional studies and therapeutic target identification. Additionally, we employed single-cell RNA sequencing (scRNA-seq) to analyze gene expression at the single-cell level within osteosarcoma tissues. Through clustering analysis, metabolic profiling, and pseudotime analysis, we explored the heterogeneity, metabolic characteristics, and developmental trajectories of osteosarcoma cells. The analyses of differential expression and enrichment revealed numerous genes and pathways exhibiting markedly abnormal expression patterns in osteosarcoma, which are closely associated with biological processes including malignant proliferation, invasion, and metastasis.

Based on the analyses, we constructed a risk signature model for osteosarcoma patients using the TARGET-OS cohort. This model integrates core genes identified through WGCNA, marker genes derived from single-cell analyses, and differentially expressed genes. Optimal prognostic gene combinations were selected using Cox regression and LASSO regression analyses. The risk signature model demonstrated robust predictive efficacy across three independent cohorts, promising to provide valuable support for the prognostic assessment and individualized treatment of osteosarcoma patients. To further validate the clinical relevance of this risk signature model, we conducted comprehensive biological and immunological analyses. Through the comparison of variations in gene expression, clinical and pathological characteristics, and the extent of immune cell infiltration among various risk categories, we observed that the group identified as high-risk demonstrated worse prognostic indicators on multiple fronts. These findings not only further validate the effectiveness of the risk signature model but also reveal potential mechanisms underlying poor prognosis in osteosarcoma patients.

In summary, this study systematically analyzes gene expression in osteosarcoma, elucidating its pathogenesis and metabolic characteristics, and constructs a risk signature model for osteosarcoma patients based on the TARGET-OS cohort. This model provides significant support for prognostic evaluation and individualized treatment, laying the groundwork for further functional studies and therapeutic target identification. The findings contribute to a deeper understanding of the pathogenesis of osteosarcoma and offer new avenues for developing treatment strategies.

## 2 Materials and methods

### 2.1 Data acquisition and preprocessing

We obtained osteosarcoma or healthy control tissue data from three sources: (1) 88 cases of bulk RNA-seq data from osteosarcoma within the TARGET-OS dataset, downloaded via the “TCGAbiolinks” R package; (2) 395 cases of bulk RNA-seq data from healthy control tissues (muscle and bone tissues), downloaded from the Genotype-Tissue Expression (GTEx, www.gtexportal.org/home/index.html) portal (20); (3) three collections of data were obtained from the Gene Expression Omnibus (GEO) database utilizing the “GEOquery” R package: GSE21257, which includes 53 cases of bulk RNA-seq data related to osteosarcoma; GSE16091, comprising 34 cases of bulk RNA-seq data concerning osteosarcoma; and GSE152048, consisting of 9 cases of single-cell RNA-seq (ScRNA-seq) data pertaining to osteosarcoma (21).

Leveraging the Combat function available in the “sva” package, we combined the TARGET-OS and GTEx datasets. Following this, the expression data underwent normalization to the Transcripts Per Kilobase of exon model per Million mapped reads (TPM) format. Any patient records with incomplete information were excluded. All data used in this study were sourced from public databases that allow unrestricted downloading and reuse. We ensured that all our analyses complied with relevant regulations, thus obviating the need for additional ethical approval.

### 2.2 Single-cell sequencing data analysis

The “Seurat” package (version 3.1.5; http://satijalab.org/seurat/) was utilized within the R software environment (version 3.6.1) to analyze the raw output data for each sample individually. Cells with fewer than 300 expressed genes or those with mitochondrial genes representing more than 10% of the total expressed genes were excluded from the analysis. Moreover, potential doublets (and, to a lesser degree, higher-order multiplets) that appeared during the encapsulation process or as pairs of undissociated cells during sample preparation were removed using the “DoubletFinder” package (version 2.0.2) in R. To address batch effects and integrate the various samples, the “harmony” R package was employed. Violin plots were created to illustrate the number of genes and transcripts identified in each sample. To pinpoint genes exhibiting significant intercellular variation in expression, we calculated both expression differences and mean expression levels across different cell subpopulations. For reducing dimensionality, we used the Uniform Manifold Approximation and Projection (UMAP) algorithm to explore the distribution of cell subpopulations within each sample (22). Additionally, Principal Component Analysis (PCA) was employed to differentiate cell subpopulations at a resolution of 0.6, with UMAP plots facilitating the visualization of the distribution of each subpopulation and their variations across different samples. By leveraging the composition of marker genes, we annotated major cell types using the “SingleR” package and visualized the expression patterns of characteristic genes for each cell subpopulation.

The “scMetabolism” R package was employed to evaluate the metabolic levels within specific cell clusters. Bubble plots were created to represent the highly activated metabolic processes across different cell clusters, whereas box plots were used to show the activity of four significantly active metabolic pathways in osteoblasts. Subsequently, we conducted unsupervised clustering analysis with the “ConsensusClusterPlus” R package, based on the metabolic levels of upregulated pathways in osteoblastic cells. The ideal number of clusters was established by finding the minimum median Proportion of Ambiguous Clustering (PAC) value. Subsequently, we utilized the “scMetabolism” R package once again to evaluate the metabolic levels of cells within each cluster. Utilizing the FindMarkers function from the “Seurat 4.4” R package, we pinpointed genes with high expression levels in cells of Cluster 1. These identified genes were then analyzed through Over Representation Analysis (ORA, which includes GO and KEGG) as well as Gene Set Enrichment Analysis (GSEA, specifically gseKEGG) by employing the “clusterProfiler” R package to assess functional enrichment.

We isolated the osteoblastic cell subpopulation and subjected it to further dimensionality reduction and clustering. Volcano plots were employed to visualize significant genes within each subpopulation. CytoTRACE2 was utilized to predict the cellular potential and absolute developmental potential of each subpopulation. Additionally, we conducted pseudotime analysis of the subpopulations using monocle2. Lastly, we examined the changes in upregulated metabolic pathways in osteoblastic cells over pseudotime.

### 2.3 Differential expression analysis and enrichment analysis

By employing the “limma” R package, we discovered differentially expressed genes (DEGs) from the merged TARGET-OS and GTEx datasets, with the selection criteria set as |logFC| > 1 and adj.p.Val < 0.05, comparing cancerous tissues to normal tissues. Visualization of these results was achieved through the generation of volcano plots and heatmaps. Following this, an analysis of Gene Ontology (GO) was carried out on the DEGs, showcasing the five most significant pathways categorized under Biological Process (BP), Cellular Component (CC), and Molecular Function (MF). In addition, a Kyoto Encyclopedia of Genes and Genomes (KEGG) analysis was executed on the DEGs, identifying the 20 pathways that exhibited the greatest statistical significance.

### 2.4 Weighted gene co-expression network analysis

We initially employed Gene Set Variation Analysis (GSVA) to assess the scores of four highly activated metabolic pathways across individual samples within the combined TARGET-OS and GTEx datasets. Subsequently, WGCNA was utilized to identify genes associated with osteosarcoma metabolism. Initially, a gene co-expression network was constructed. By calculating the scale-free topology index under varying soft-thresholding powers (β), we determined the optimal β value for network construction. Next, we employed a hierarchical clustering algorithm to partition the network into multiple co-expression modules, each represented by a distinct color. To further investigate the relationship between these modules and the four metabolic pathways, we computed the correlations between module eigengenes and the four metabolic pathways, generating a module eigengene heatmap. Modules with absolute correlation coefficients greater than 0.3 and statistical significance were prioritized for further analysis. In these modules, we explored the relationship between module membership and the importance of genes to pinpoint hub genes. Ultimately, we performed GO enrichment analysis on the key genes found within these modules.

### 2.5 Risk signature based on osteosarcoma patients from the TARGET-OS cohort

To establish a risk signature using the TARGET-OS cohort as the training set, we first identified the intersection of core genes from WGCNA analysis, marker genes derived from single-cell analysis, and DEGs, and visualized this intersection using a Venn diagram. Next, we carried out a univariate Cox regression analysis focused on the intersecting genes to evaluate their link to patient survival. Afterward, we executed Least Absolute Shrinkage and Selection Operator (LASSO) regression analysis on those same intersecting genes, identifying the best prognostic genes using the optimal parameter λ. The formula was utilized to calculate the score for each patient:


$$Risk\;score=\sum_{n}^{i=1}\left[GeneExp_{i}*Coefficient_{i}\right]$$

According to the median score, samples from patients with osteosarcoma were divided into groups of high and low risk. We used GSE21257 and GSE16091 for validation purposes. In the end, Kaplan-Meier curves were used to depict the survival outcomes for each group across three separate cohorts. Furthermore, we plotted time-dependent receiver operating characteristic (ROC) curves to evaluate the discriminatory power for patient survival at various time points (1, 3, and 5 years).

### 2.6 Further analysis of the model

We first analyzed the differences in gene expression and clinical pathological features between the two risk groups. Following this, the “limma” R package was utilized to identify DEGs, and GSEA was performed to illustrate the KEGG pathways that were upregulated and downregulated in connection with these DEGs. Next, we retrieved cancer hallmarks from the Molecular Signatures Database (MSigDB, https://www.gsea-msigdb.org/gsea/msigdb) and compared the scores of different cancer hallmark pathways across the groups using GSVA.

We utilized the CIBERSORT algorithm via the “IOBR” R package to calculate the levels of immune cell infiltration, subsequently comparing these levels across the two risk categories. Afterward, we evaluated the variations in the expression of immune factors and estimated the therapeutic impacts of immune checkpoint inhibitors, relying on Tumor Immune Dysfunction and Exclusion (TIDE) scores. Next, we examined the differences in pro-tumor immune cells, particularly Cancer-Associated Fibroblasts (CAF) and Myeloid-Derived Suppressor Cells (MDSC), between the two groups. In conclusion, we employed the “OncoPredict” R package to assess the drug responsiveness of each group.

### 2.7 Cell culture

In this study, the following cell lines were utilized for *in vitro* experiments: Normal Human Osteoblast (NHOST) Cells, MG63, SAOS2, U2OS, and HOS (from the Chinese Academy of Sciences Cell Bank). Among these, NHOST served as the normal control cell line, while the others were classified as tumor cell lines. NHOST was cultured in OGM BulletKit (Lonza, Switzerland) medium; MG63 was maintained in Minimum Essential Medium (MEM, Hyclone, USA); SAOS2 and U2OS were cultured using McCoy’s 5A medium (Hyclone, USA); and HOS was grown in Dulbecco’s Modified Eagle Medium (DMEM, Hyclone, USA). All culture media were supplemented with 10% fetal bovine serum (FBS, Hyclone, USA), and 1% penicillin-streptomycin mixed solution (Keygen, China) was added to inhibit bacterial growth. All cells were incubated in a humidified environment at 37°C with 5% CO_2_ to maintain logarithmic growth.

### 2.8 Transfection

Transfection experiments were performed on the MG63 and SAOS2 cell lines, utilizing siRNA (Sangon, China) for transient transfection to knock down the COL5A1 gene. A negative control (NC) was used as a reference group. Initially, cells were seeded in six-well plates and allowed to reach 80% confluency. A suitable amount of Opti-MEM reduced serum medium (Thermo, USA) was used to dissolve Lipofectamine 3000 (Thermo, USA) and siRNA, followed by a 5-minute incubation. The two solutions were then mixed and allowed to sit for 20 minutes before being added to the six-well plates. After transfection, the culture medium was replaced after 5 hours. During transfection, no antibiotics were added to any medium to avoid their effect on cell activity.

### 2.9 Total RNA extraction and RT-qPCR

Cells were digested and collected at the bottom of the tube. Trizol (Takara, Japan) was used to lyse the cells with 950 μl to inhibit RNAse activity. After 5 minutes, 150 μl of chloroform (China National Pharmaceutical Group, China) was added, and the mixture was vortexed until it resembled watermelon juice. After centrifugation for 5 minutes, the supernatant was collected and mixed with an equal volume of isopropanol (SINOPHARM, China) to precipitate RNA. The mixture was then centrifuged again for 5 minutes, retaining the precipitate, which was washed with 1 ml of 75% ethanol or anhydrous ethanol, and thoroughly dried. All operations were conducted to ensure the absence of RNAse contamination, and the RNA concentration, DNA contamination, and protein contamination were measured post-extraction.

Subsequently, the PrimeScript RT kit (TaKaRa, Japan) was employed to eliminate genomic DNA. Based on the manufacturer’s recommendations and the measured RNA concentration, an appropriate liquid mix was prepared to remove DNA. Reverse transcription was then performed to generate cDNA. Real-time quantitative PCR analysis was conducted based on the SYBR GreenER Supermix (TaKaRa, Japan) instructions, with all samples and reagents pre-mixed and analyzed on a Roche480 PCR system (Roche, Switzerland). Each group included three technical replicates, with β-actin serving as the internal control.

### 2.10 Cell counting kit-8

Twenty-four hours post-transfection, cells were seeded into a 96-well plate (4000 cells/well) and allowed to adhere, with three technical replicates set for each group. CCK8 reagent (KeyGEN, China) was pre-mixed with the culture medium according to the manufacturer’s instructions, resulting in a final volume of 100 μl per well. The plates were then shielded from light and placed in the incubator. After 1.5 hours, the cells were analyzed using a spectrophotometer at a wavelength of 450 nm, with measurements repeated at various time points.

### 2.11 Statistical analysis

In this research, a survival analysis was carried out utilizing the Kaplan-Meier technique, while the log-rank test was applied to assess and compare the survival curves of individuals categorized into high-risk and low-risk groups. The area under the receiver operating characteristic (ROC) curve (AUC) was determined, with an AUC exceeding 0.6 interpreted as a sign of reliable test performance. A p-value below 0.05 was considered statistically significant in all evaluations. All statistical procedures were executed using R software (version 4.3.1).

## 3 Results

### 3.1 Single-cell sequencing data analysis

**Figure 1:**
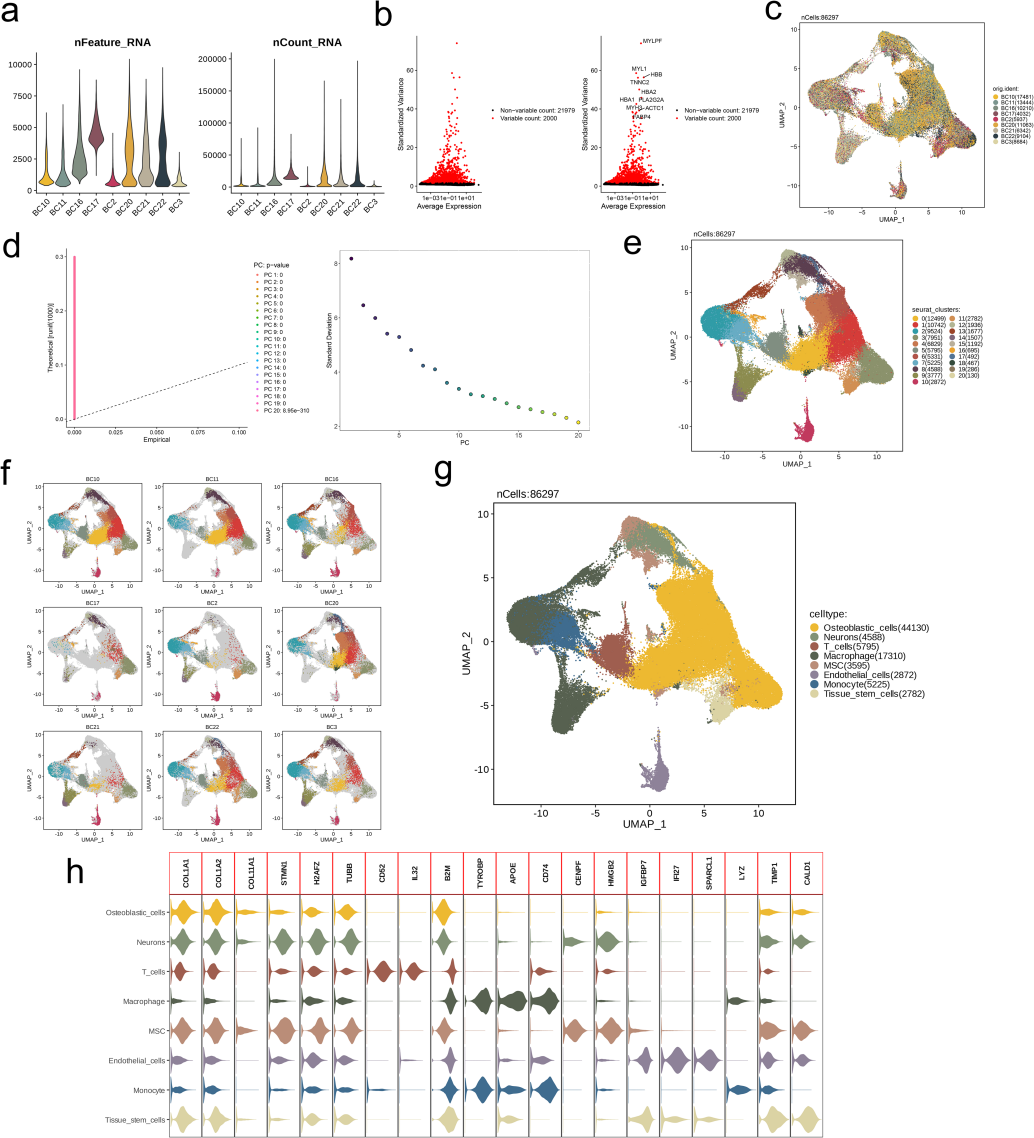
Identification of 8 cell clusters with diverse annotations revealing high cellular heterogeneity in OS based on single-cell RNA-seq data. **(A)** After quality control of scRNA-seq, 86297 core cells were identified. **(B)** The variance diagram shows the variation of gene expression in all cells of OS. The red dots represent highly variable genes and the black dots represent non-variable genes. **(C)** UMAP showed a clear separation of cells in OS. **(D)** PCA identified the top 20 PCs at p<0.05. **(E)** The umap algorithm was applied to the top 20 PCs for dimensionality reduction, and 21 cell clusters were successfully classified. **(F)** Classification of cell clusters in each sample. **(G)** All 8 cell clusters in OS were annotated with SingleR and CellMarker according to the composition of marker genes. **(H)** Expression levels of marker genes for each cell cluster.

We conducted quality control by comparing the number of detected genes and transcripts across the selected nine samples (**Figure 1A**). Analysis of gene expression differences and average expression levels in various cell subpopulations revealed significant expression differences for MYLPF, MYL1, HBB, TNNC2, HBA2, HBA1, PLA2G2A, MYH3, ACTC1, and FABP4 across these subpopulations (**Figure 1B**). The UMAP plot illustrated a mathematically uniform distribution of cells from the nine samples (**Figure 1C**). Subsequently, we applied Principal Component Analysis (PCA) to cluster the cell subpopulations. The clustering yielded 21 distinct cell subpopulations, which exhibited the lowest standard deviation and statistically significant results (**Figure 1D**). The UMAP plot also demonstrated effective clustering of the different cell subpopulations (**Figure 1E**). This clustering approach effectively distinguished between cell populations across the various samples (**Figure 1F**). Next, we identified and annotated the cell subpopulations based on biological classification, resulting in Osteoblastic cells (44130), Neurons (4588), T cells (5795), Macrophages (17310), MSC (3595), Endothelial cells (2872), Monocytes (5225), and Tissue stem cells (2782) (**Figure 1G**). Finally, we visualized the marker genes for each cell subpopulation (**Figure 1H**).

### 3.2 Metabolic analysis of single-cell subpopulations

**Figure 2:**
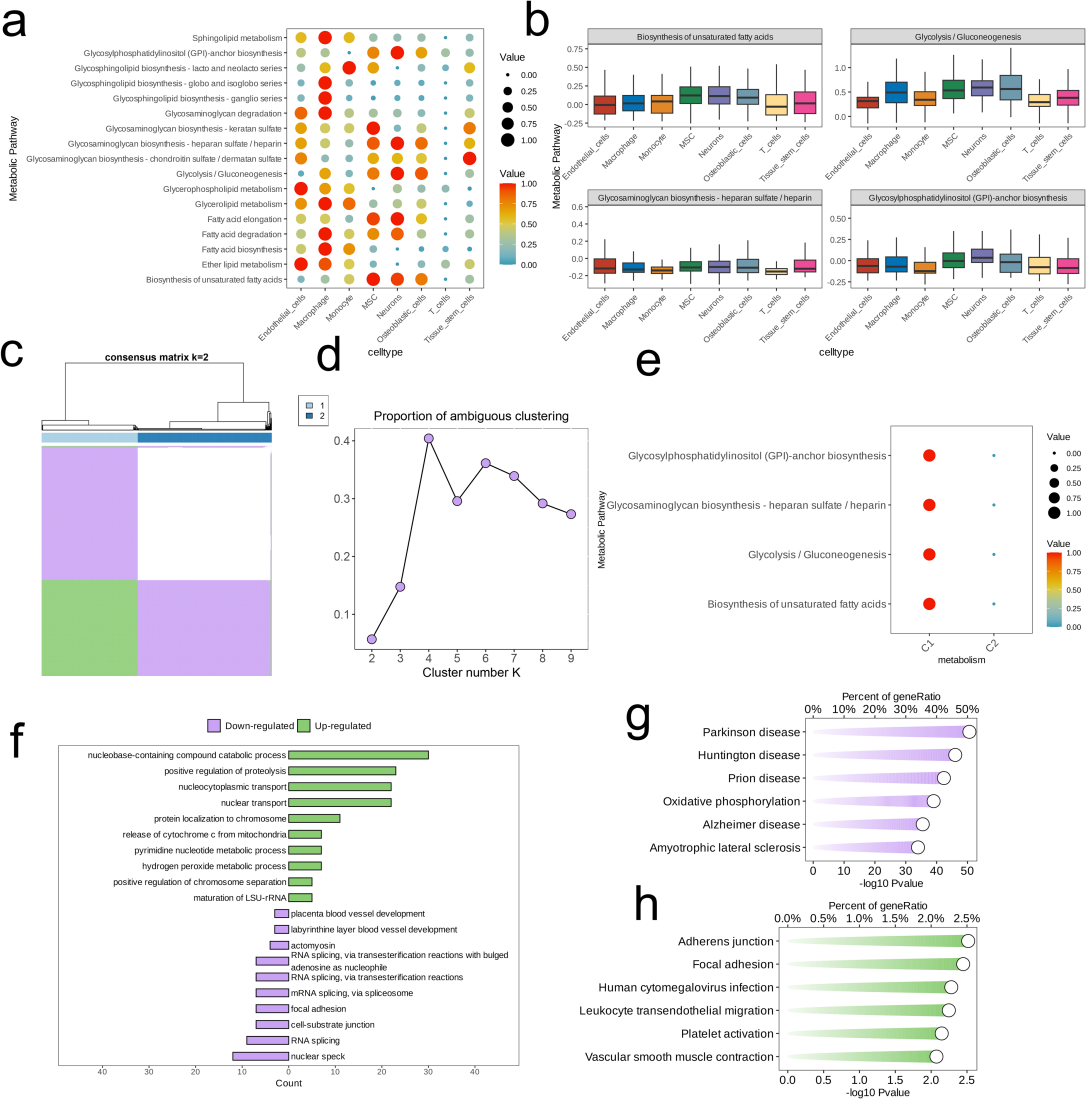
Identification of cell clusters with highly activated metabolism activities in OS at the single cell level. **(A)** The highly activated metabolic process of in each cell cluster revealed by the “scMetabolism” R package. **(B)** Boxplots showing the activities of four highly activated metabolic pathways in osteoblastic cells. **(C)** Consensus matrix(k=2). **(D)** The proportion of ambiguous clustering (PAC) score, a low value of PAC implies a flat middle segment, allowing conjecture of the optimal k (k = 2) by the lowest PAC. **(E)** Two distinct metabolism patterns of OS at the single-cell level unraveled by the unsupervised clustering. **(F–H)** Barplot reveals the dysregulated GO-BP terms **(F)** and KEGG pathways **(G, H)** in OS cells with highly activated metabolism activities.

There is heterogeneity in the activity of various metabolic pathways across different cell subpopulations. Glycerophospholipid metabolism is notably activated in Endothelial cells, Monocytes, and Macrophages. Interestingly, we observed that the metabolic pathways of Glycosylphosphatidylinositol (GPI)-anchor biosynthesis, Glycosaminoglycan biosynthesis—specifically heparan sulfate/heparin, Glycolysis/Gluconeogenesis, and Biosynthesis of unsaturated fatty acids are highly activated in Osteoblastic cells, Neurons, and Mesenchymal Stem Cells (MSC). The overall activation levels of metabolic pathways also display heterogeneity across the subpopulations, with the Macrophage subpopulation exhibiting the highest number of active metabolic pathways, while all pathways in T cells remain inactive. In the Osteoblastic cell subpopulation, the aforementioned four metabolic pathways are significantly activated (**Figure 2A**). We presented a box plot to illustrate the activation levels of these pathways across different cell subpopulations, showing that their activation in Osteoblastic cells is generally higher than in the other subpopulations (**Figure 2B**).

Subsequently, we performed consensus clustering, which exhibited high cohesion and low coupling when the number of clusters (k) was set to 2 (**Figure 2C**). At this point, the PAC value was at its minimum (**Figure 2D**). The differences in activation levels across the four metabolic pathways were examined between the two clusters, showing that cluster 1 exhibits notably greater pathway activation than cluster 2 (**Figure 2E**).

Next, we examined the highly expressed genes in cluster 1 and conducted an Over-Representation Analysis (ORA). The results indicated that the cells in cluster 1 are enriched in pathways and biological processes related to cellular metabolism and catabolism (nucleobase-containing compound catabolic process, pyrimidine nucleotide metabolic process), apoptosis and cell death (positive regulation of proteolysis, release of cytochrome c from mitochondria), and nucleocytoplasmic transport (nucleocytoplasmic transport, nuclear transport, protein localization to chromosome). In contrast, the cells found in cluster 1 exhibited a downregulation in pathways or biological processes related to RNA splicing and processing (involvement of actomyosin in RNA splicing through transesterification reactions involving bulged adenosine as a nucleophile, splicing of RNA via transesterification reactions, and mRNA splicing through the spliceosome), cell adhesion and junctions (including focal adhesion and cell-substrate junctions), as well as nuclear structure and function (nuclear speck) (**Figure 2F**). The results of the KEGG analysis revealed that the cells within cluster 1 exhibited a decrease in oxidative phosphorylation while demonstrating an increase in pathways associated with adherens junctions, focal adhesion, leukocyte transendothelial migration, platelet activation, and vascular smooth muscle contraction (**Figures 2G-H**).

### 3.3 Analysis of osteoblastic cell subpopulations

**Figure 3:**
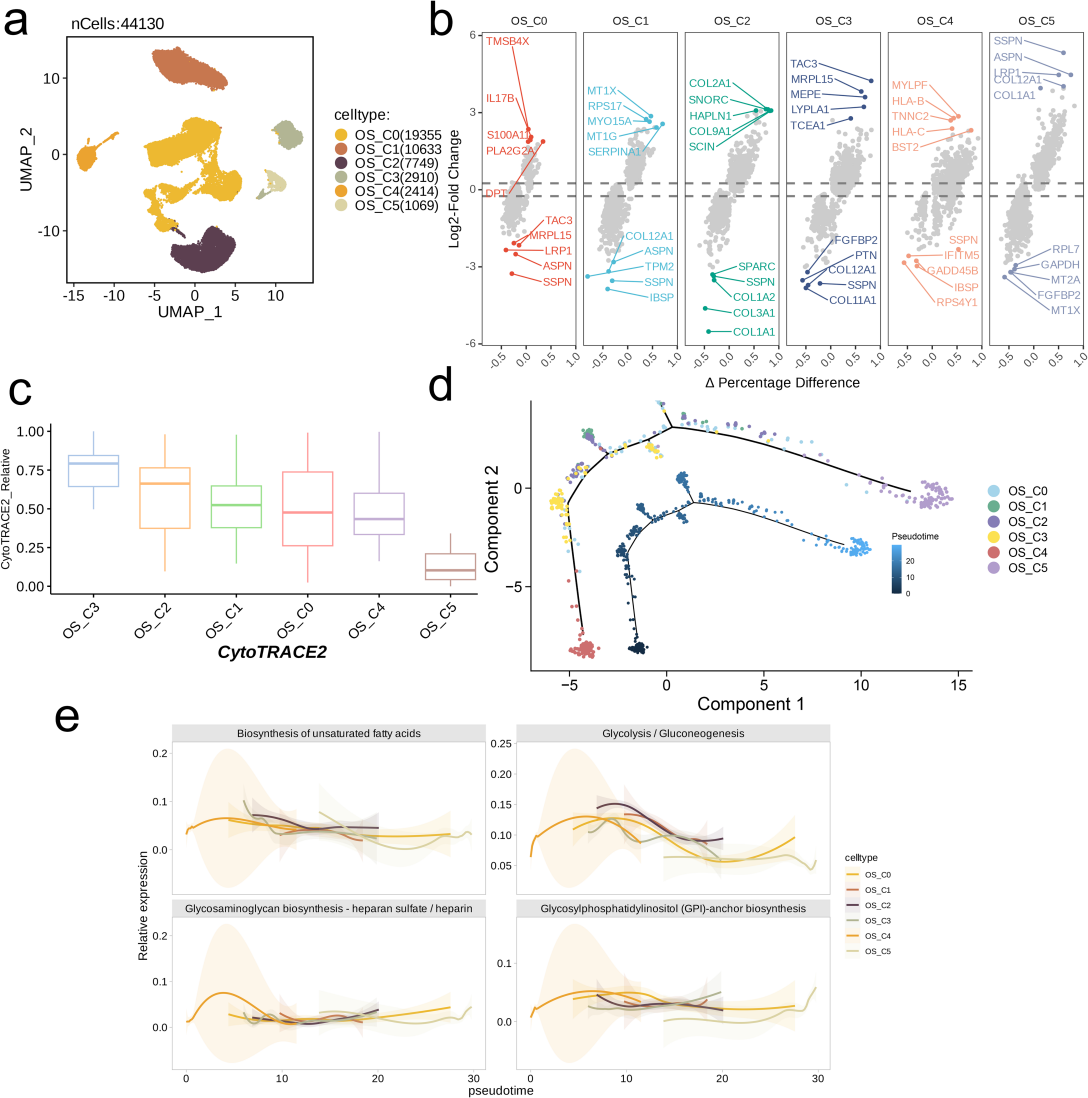
Trajectory analysis of OS cell subsets with distinct differentiation patterns. **(A)** UMAP visualization of the subsets of osteoblastic cells. **(B)** Volcano plots showing the celltype-specific markers of each subset. **(C)** Boxplots showing the predicted cellular potency and absolute developmental potential of osteoblastic cell subset. **(D)** Trajectory analysis revealed cell subsets of osteoblastic cells with distinct differentiation states. **(E)** The variations of metabolic pathway activities along with the pseudotime.

We performed dimensionality reduction clustering on 44,130 Osteoblastic cells, identifying a total of six distinct subpopulations (**Figure 3A**). The volcano plot revealed heterogeneity in the top five highly expressed or lowly expressed genes among the subpopulations. Notably, the gene expression profile of the C5 subpopulation was significantly different from that of the other subpopulations. Specifically, SSPN was markedly underexpressed in all other subpopulations but exhibited the opposite trend in C5; ASPN, COL12A1, COL1A1, and LRP1 were significantly underexpressed in certain subpopulations but were upregulated in C5; conversely, MT1X was lowly expressed in C5 but highly expressed in C1 (**Figure 3B**). Results from CytoTRACE2 indicated that C3 possesses the highest differentiation potential, while C2, C1, C0, and C4 displayed similar potentials. C5 had the lowest differentiation potential (**Figure 3C**). The results from pseudo-time analysis were consistent, with C4 located at the starting point of the pseudo-time trajectory, followed by C3. As pseudo-time increased, differentiation into C2, C1, and C0 occurred, with C5 positioned at the endpoint of the pseudo-time path (**Figure 3D**). Interestingly, despite C4 theoretically appearing earlier in the pseudo-time framework than C3, its differentiation potential was not as robust as that of C3. The activity levels of the four metabolic pathways also mirrored these findings. The pathways in C4, C3, and C0 were activated earlier and with greater intensity, while C1 and C2 followed closely behind. C5 exhibited the latest activation and the lowest intensity of pathway activation (**Figure 3E**).

### 3.4 Differential expression and enrichment analysis

**Figure 4:**
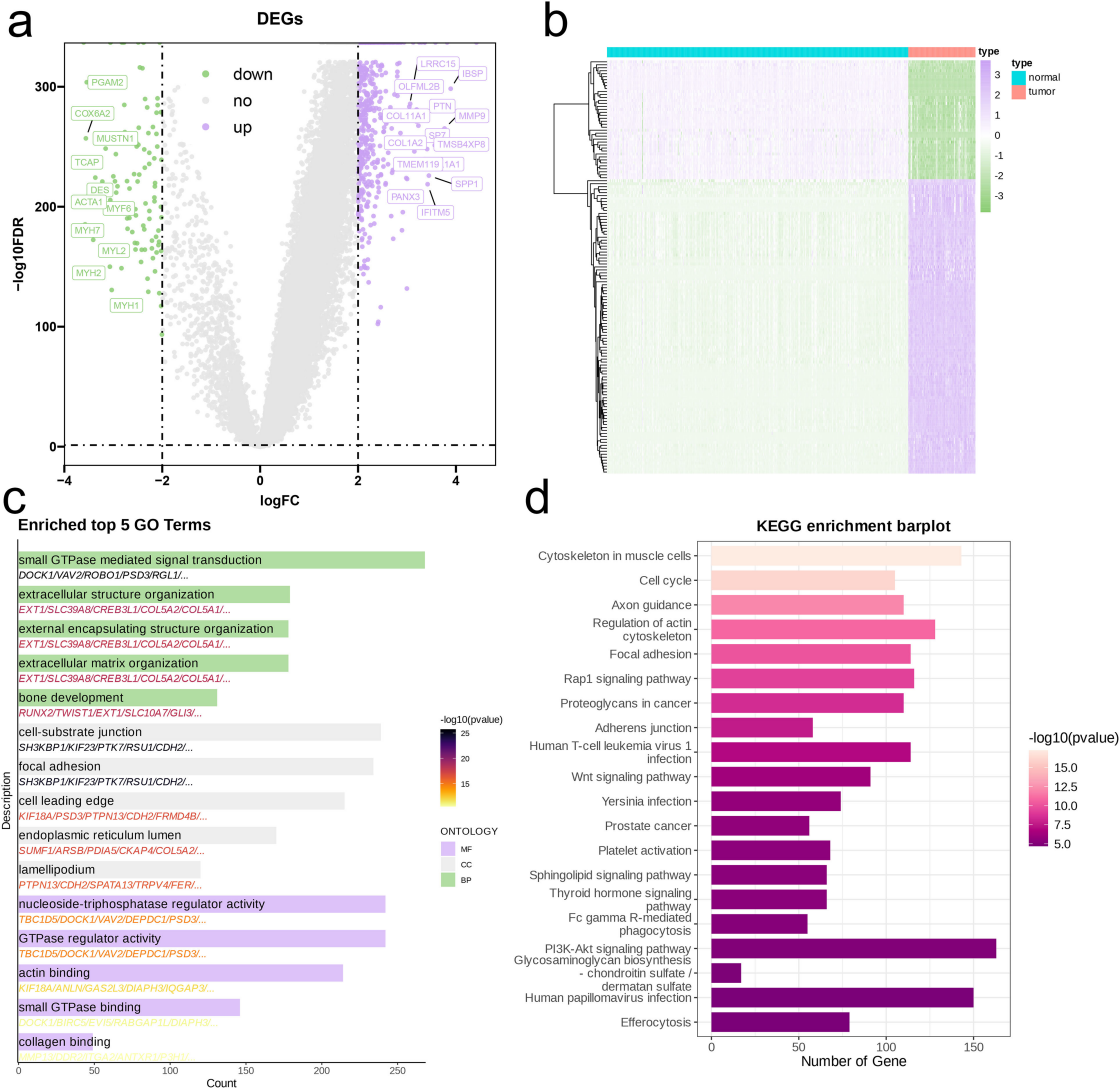
Identification and functional enrichment analysis of DEGs between OS patients and controls. **(A)** Volcano plot of DEGs between OS and control in the merged cohort of TARGET-OS and GTEx. P<0.05 and |log2FoldChange|>1 were identified as significant DEGs. **(B)** b Heatmap of DEGs. **(C, D)** Barplots of the BP, CC, MF **(C)**, and KEGG pathways **(D)** of DEGs.

We conducted a differential expression analysis on the merged bulk RNA sequencing dataset, visualizing the results through volcano plots and heatmaps. Overall, the number of genes expressed at higher levels in the tumor group was significantly greater (**Figures 4A, B**). Gene Ontology (GO) analysis of the DEGs indicated that tumor tissues were primarily enriched in processes related to cell signaling and regulation (small GTPase mediated signal transduction, nucleoside-triphosphatase regulator activity, GTPase regulator activity), cell structure and motility (extracellular structure organization, external encapsulating structure organization, extracellular matrix organization, cell-substrate junction, focal adhesion, cell leading edge, lamellipodium), bone and connective tissue development (bone development, collagen binding), and intracellular structure and function (endoplasmic reticulum lumen, actin binding) (**Figure 4C**). The results from the KEGG pathway analysis were consistent, showing that tumor tissues were predominantly enriched in pathways related to cytoskeleton and structure (Cytoskeleton in muscle cells, Regulation of actin cytoskeleton, Focal adhesion, Adherens junction), cell cycle and proliferation (Cell cycle), signaling and regulation (Rap1 signaling pathway, Wnt signaling pathway, PI3K-Akt signaling pathway, Sphingolipid signaling pathway), as well as Proteoglycans in cancer (**Figure 4D**).

### 3.5 WGCNA

**Figure 5:**
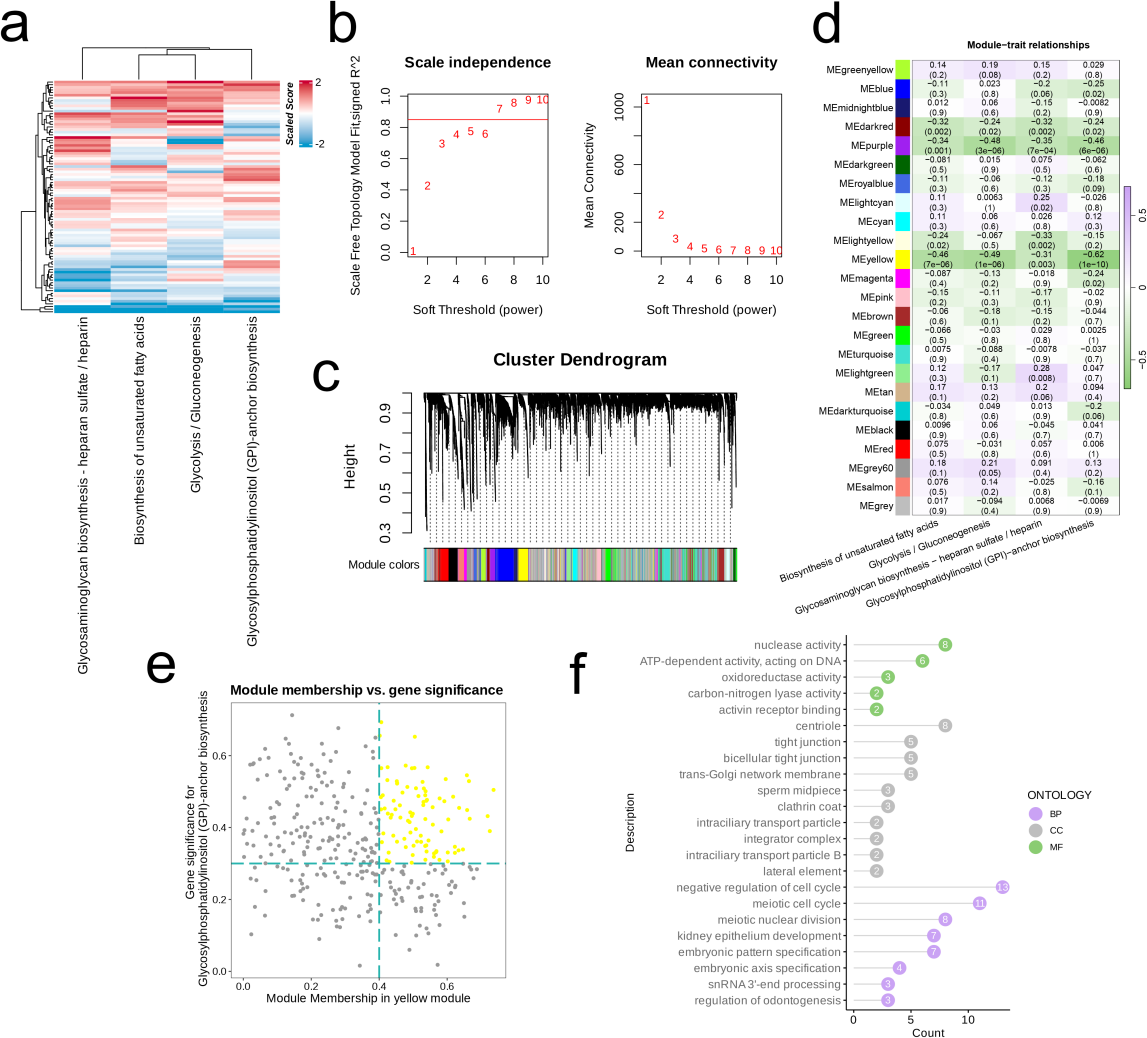
Metabolism-related genes were screened by WGCNA. **(A)** Heatmap of the four highly activated metabolic pathways. **(B)** Analysis of the scale-free index for various soft-threshold powers (β). **(C)** Cluster dendrogram of the coexpression modules. Each color indicates a co-expression module. **(D)** Module-trait heatmap displaying the correlation between module eigengenes and clinical traits. **(E)** Correlation between module membership and gene significance in the yellow modules. Dots in colors were regarded as the hub genes of the module. **(F)** The top enriched GO terms of the hub genes of the module.

We first employed GSVA to evaluate the scores of four highly activated metabolic pathways across samples in the merged dataset (**Figure 5A**). Subsequently, we determined the optimal soft threshold (β) for constructing a scale-free network by analyzing scale independence and mean connectivity (**Figures 5B, C**). We calculated the correlation between module characteristics and the four metabolic pathways, revealing a significant negative correlation of the MEyellow module with all four pathways (R < -0.3, p < 0.01, **Figure 5D**). Next, we performed further filtering of the genes within this module to identify core genes (**Figure 5E**). The Gene Ontology (GO) analysis of these core genes indicated significant enrichment in several biological functions (**Figure 5F**), including nucleic acid enzymatic activity and DNA metabolism (nuclease activity, ATP-dependent activity acting on DNA, snRNA 3’-end processing), redox reactions and metabolism (oxidoreductase activity, carbon-nitrogen lyase activity), signaling and receptor binding (activin receptor binding), cellular structure and connections (centriole, tight junction, bicellular tight junction, lateral element), intracellular transport and membrane structures (trans-Golgi network membrane, clathrin coat, intraflagellar transport particle, integrator complex, intraflagellar transport particle B), as well as cell cycle regulation (negative regulation of cell cycle).

### 3.6 Risk signature for osteosarcoma patients based on the TARGET-OS cohort

**Figure 6:**
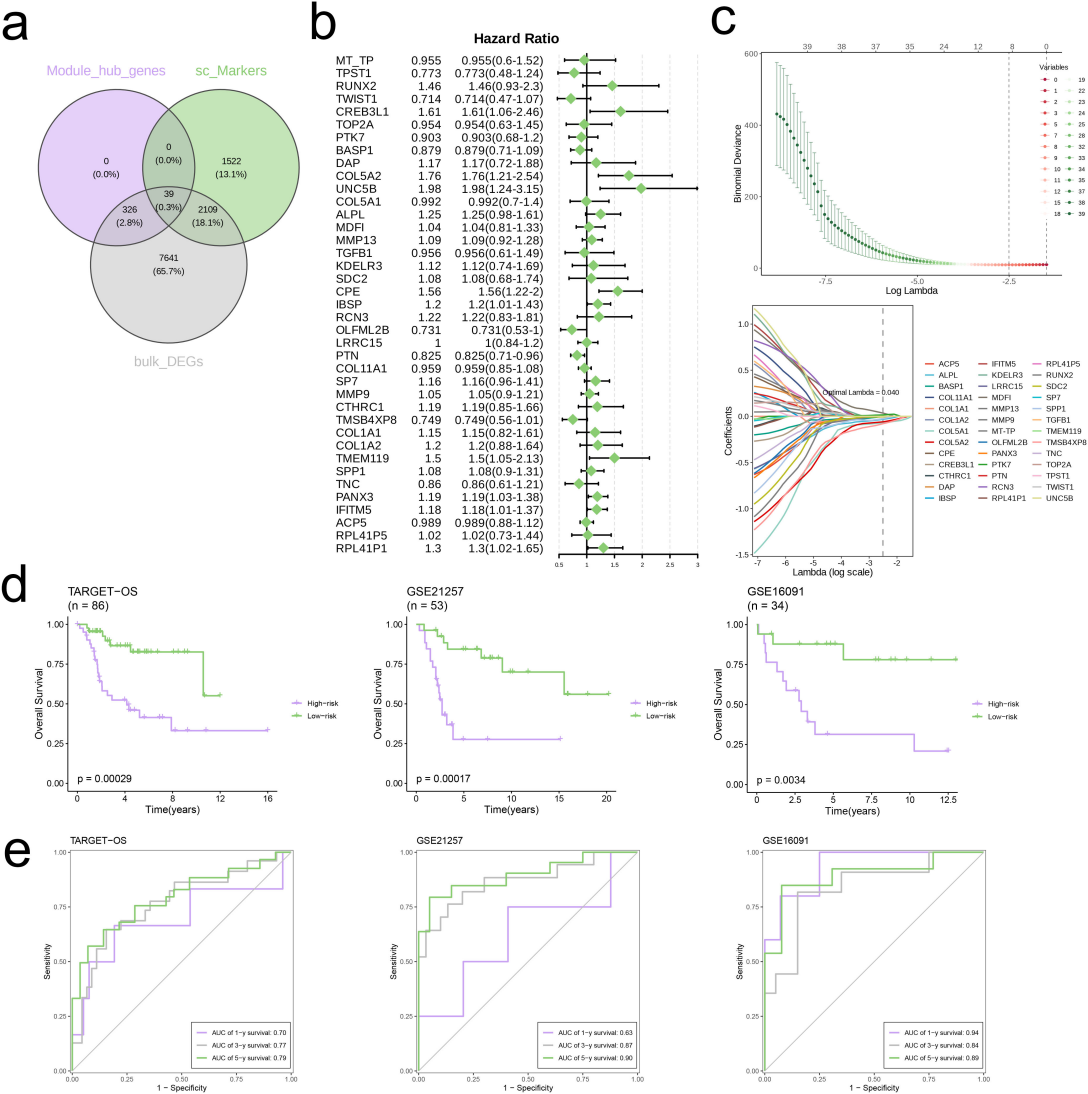
Construction of risk signature in the TARGET-OS cohort. **(A)** Venn diagram analysis of hub genes of modules, single-cell markers, and DEGs from TARGET-OS bulk cohort. **(B)** Univariate cox regression analysis of 39 genes in TARGET-OS cohort. **(C)** The selection of prognostic genes based on the optimal parameter λ that was obtained in the LASSO regression analysis. **(D)** K-M curves displayed survival outcomes of patients in two risk groups from the three cohorts. **(E)** Time-dependent ROC curves were drawn to assess survival rate at 1-year, 3-year, and 5-year in the three cohort.


We first identified the intersection of core genes from the WGCNA analysis, marker genes obtained from single-cell analysis, and DEGs, visualizing the results using a Venn diagram (**Figure 6A**). A univariate Cox regression analysis of the intersected genes revealed heterogeneity in their prognostic impacts (**Figure 6B**). Afterward, we conducted LASSO regression analysis on the genes that intersected (**Figure 6C**) and determined the best lambda value of 0.040, resulting in the subsequent formula for the risk score:

$$\text{Risk score}=\text{PTK}7^{*}\left(-0.015\right)+\text{COL}5\text{A}2^{*}\left(-0.108\right)+\text{COL}5\text{A}1^{*}\left(-0.122\right)+{\text{ALPL}}^{*}\left(-0.093\right)+{\text{MDFI}}^{*}\left(0.143\right)+\text{SDC}2^{*}\left(-0.012\right)+\text{TMSB}4\text{XP}8^{*}\left(-0.159\right)+\text{SPP}1^{*}\left(-0.103\right)$$

Kaplan-Meier survival curves demonstrated that the high-risk group exhibited significantly poorer survival compared to the low-risk group across three independent cohorts (AUC>0.6, **Figures 6D, E**).

### 3.7 Further analysis of the model

**Figure 7:**
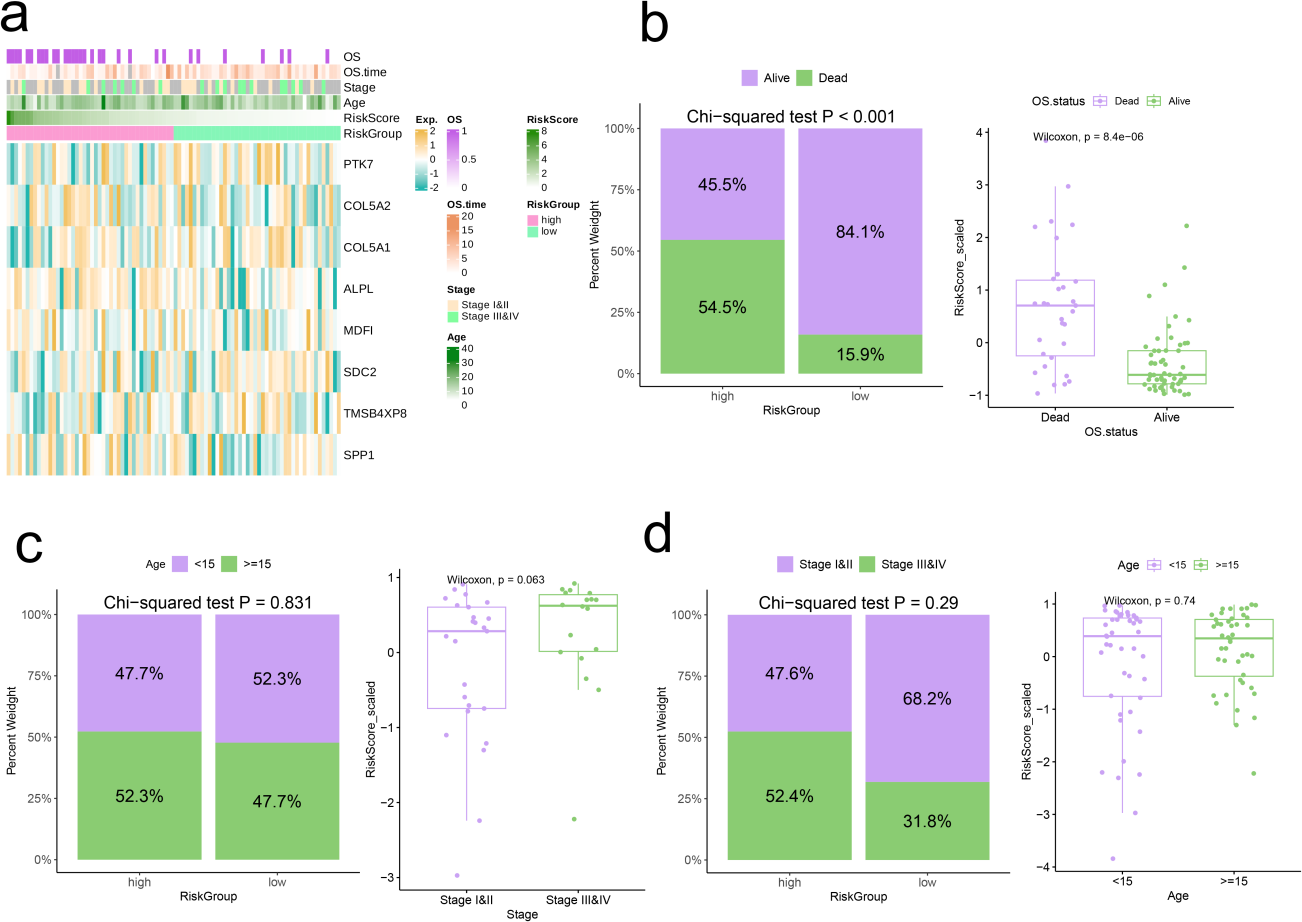
Correlation analysis of risk scores with clinical characteristics. **(A)** Heatmap of risk model and clinical characteristics. **(B-D)** Relationship between age, stage, and survival status with the analysis model.

**Figure 8:**
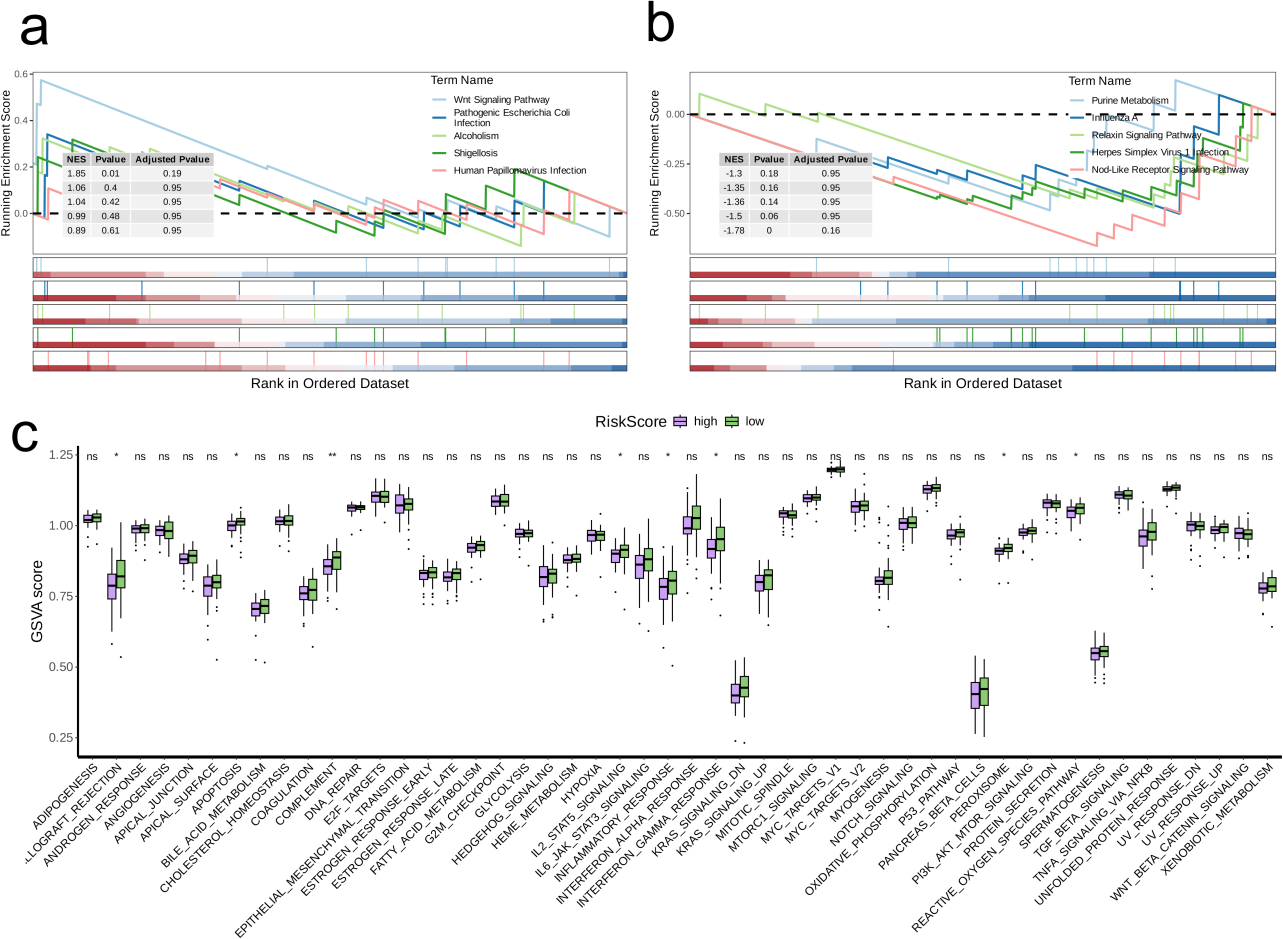
Biological characteristics between high-and low-risk groups. **(A, B)** The upregulated **(A)** and downregulated **(B)** KEGG pathways in high-risk group. **(C)** The differences of estimated GSVA scores of cancer hallmarks between high- and low-risk groups.

Heatmaps were employed to illustrate the differences in expression levels of model genes alongside clinical pathological characteristics across the two risk categories (**Figure 7A**). It is important to highlight that the high-risk category exhibited an elevated mortality rate, with patients who had passed away showing significantly increased risk scores (p < 0.05, **Figures 7C, D**). Subsequently, we pinpointed DEGs between the two risk categories and performed GSEA on these identified DEGs. The Wnt Signaling Pathway showed significant upregulation in the high-risk category (Normalized Enrichment Score (NES) = 1.85, p = 0.01, **Figure 8A**), whereas the Nod-Like Receptor Signaling Pathway was prominently upregulated in the low-risk category (NES = -1.78, p < 0.05, **Figure 8B**).

**Figure 9:**
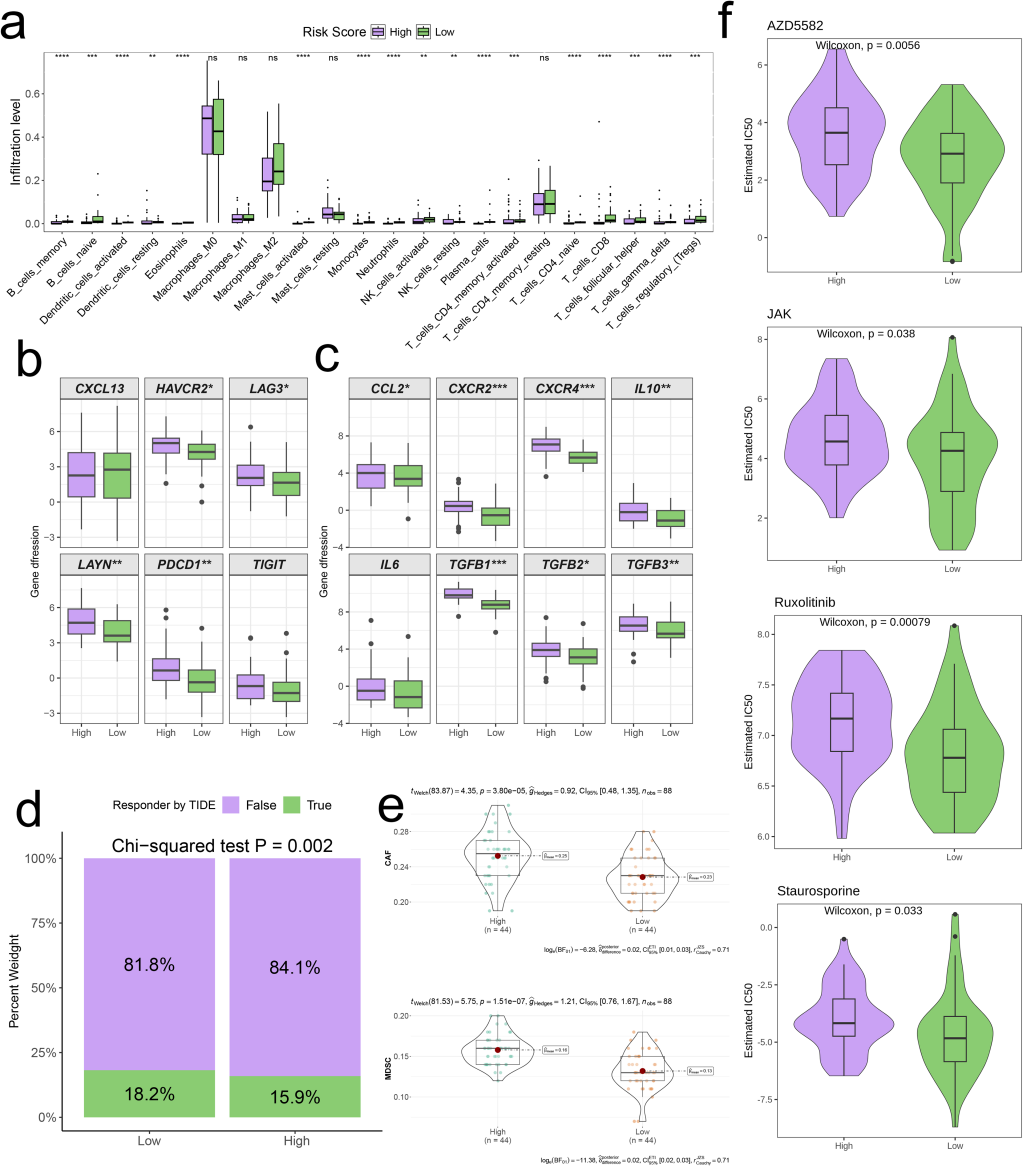
Distinct TME landscapes and therapeutic agents between high-and low-risk groups. **(A)** Box plot illustrating the distributions of 22 immune cell subsets determined by CIBERSORT between two risk groups. **(B, C)** Box plot illustrating the expression profiles of T cell exhaustion markers **(B)** and M2 polarization regulators **(C)** between two risk groups. **(D)** Stacked plot showed the distribution of predicted responders determined by the TIDE webtool between two risk groups. **(E)** Violin plot displaying the infiltration levels of CAF and MDSC between two risk groups. **(F)** Violin plot displaying the estimated IC50 of therapeutic agents between two risk groups.

Using GSVA, we assessed the scores of multiple cancer hallmark pathways between the two groups. The findings demonstrated that the high-risk group had notably lower scores in various pathways, such as Allograft Rejection, Apoptosis, Complement, IL2-STAT5 Signaling, Inflammatory Response, Interferon Gamma Response, Peroxisome, and the Reactive Oxygen Species Pathway (p < 0.05, **Figure 8C**). Additionally, we determined the levels of immune cell infiltration for both risk groups utilizing the CIBERSORT algorithm. The results indicated that the low-risk group showed significantly higher levels of immune cell infiltration across all examined immune cell types compared to the high-risk group (p < 0.01, **Figure 9A**). In particular, immune-related genes including LAYN, HAVCR2, PDCD1, LAG3, CCL2, IL6, CXCR2, TGFB1, CXCR4, TGFB2, IL10, and TGFB3 were substantially elevated in the high-risk group (p < 0.05, **Figures 9B, C**). Moreover, the TIDE scores indicated that the percentage of patients in the high-risk category classified as “True” was significantly lower than that in the low-risk category (p = 0.002, **Figure 9D**). This observation implies that patients in the high-risk group may have heightened tumor immune evasion capabilities, which can lead to variations in immunotherapy response and potentially poorer prognosis. Furthermore, the concentrations of cancer-associated fibroblasts (CAF) and myeloid-derived suppressor cells (MDSC) were significantly elevated in the high-risk group (p < 0.001, **Figure 9E**). Finally, we conducted drug sensitivity assessments, which illustrated that individuals in the high-risk group showed diminished sensitivity to several therapeutic agents, including AZD5582, JAK inhibitors, Ruxolitinib, and Staurosporine, when compared to those in the low-risk group (p < 0.05, **Figure 9F**). This suggests that the high-risk cohort may exhibit resistance to certain treatment modalities.

### 3.8 COL5A1 promotes proliferation of osteosarcoma cells

**Figure 10:**
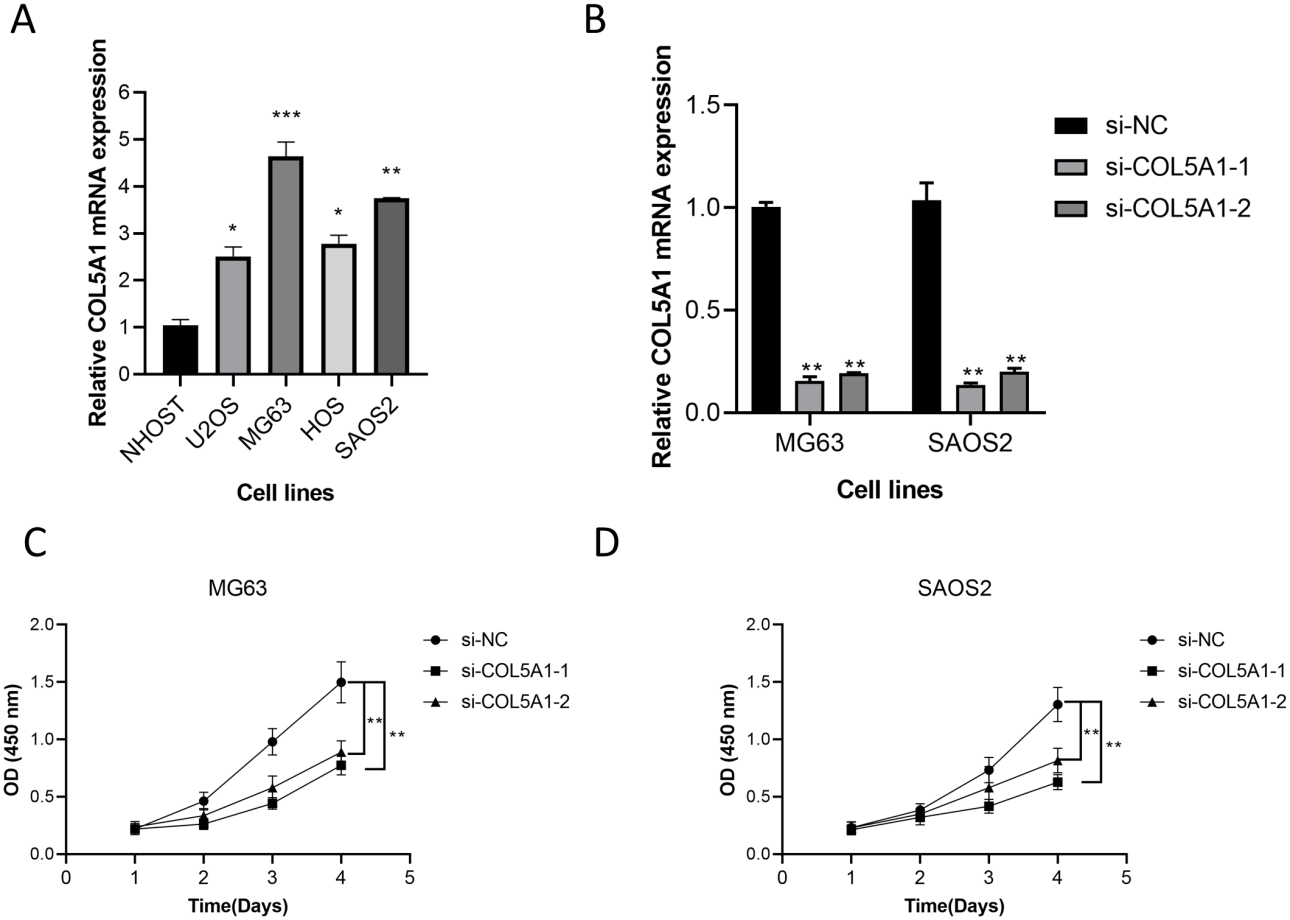
The effect of COL5A1 on osteosarcoma was verified by wet experiment. **(A)** Comparison of mRNA expression levels of COL5A1 between cell lines. **(B)** Evaluation of COL5A1 knockdown efficiency. **(C)** Changes in proliferation levels after COL5A1 knockdown in MG63 cell lines. **(D)** Changes in proliferation levels after COL5A1 knockdown in SAOS2 cell lines.

Currently, there is a lack of research on the role of COL5A1 in osteosarcoma cells; therefore, we selected this gene from our model for further experimental validation. Comparative expression analysis among the cell lines revealed that COL5A1 was significantly overexpressed in osteosarcoma cell lines (p < 0.05, **Figure 10A**). Subsequently, we performed knockdown experiments in two cell lines, demonstrating a high knockdown efficiency (p < 0.01, **Figure 10B**). Results from the CCK8 assay indicated that knockdown of COL5A1 significantly inhibited the proliferation of tumor cells (p < 0.01, **Figures 10C, D**). These findings suggest that COL5A1 plays a role in promoting osteosarcoma cell proliferation.

## 4 Discussion

Osteosarcoma, also known as osteogenic sarcoma, is a malignant tumor that originates from mesenchymal bone tissue and is characterized by the direct production of osteoid or bone matrix by tumor cells (23). The most frequently occurring primary malignant bone tumor primarily impacts adolescents between the ages of 10 and 20, as well as individuals older than 65, showing a slightly greater prevalence in males compared to females (24). The tumor primarily affects long bones, such as the distal femur and proximal tibia, and exhibits certain hereditary predispositions and geographic variations. Osteosarcoma is highly malignant, and its prognosis is often poor. Various factors influence the prognosis, but early detection and standardized comprehensive treatment are crucial for improving patient survival rates. In spite of the recent progress made in surgical, chemotherapy, and radiotherapy techniques, the five-year survival rate for patients with osteosarcoma has seen little improvement, posing considerable challenges for treatment.

GPI-anchor biosynthesis is a vital post-translational modification process that involves anchoring non-transmembrane proteins to the outer leaflet of the cytoplasmic membrane, thereby participating in various biological processes, including signal transduction, cell adhesion, transport, and metabolism (25, 26). The creation of glycosaminoglycans, particularly heparan sulfate/heparin, is a multifaceted process that requires the enzymatic activity of several different enzymes. Glycosaminoglycans belong to a group of linear polysaccharides made up of repeating units of disaccharides. Acetylated heparan sulfate/heparin serves an important structural and regulatory function within the extracellular matrix and on cellular surfaces, engaging in a variety of biological activities, including cell adhesion, signal transduction, coagulation, and angiogenesis (27). Glycolysis and gluconeogenesis represent core pathways in energy metabolism (28, 29). Glycolysis involves the breakdown of glucose into pyruvate, generating ATP, while gluconeogenesis refers to the conversion of non-carbohydrate precursors into glucose or glycogen (30). Both pathways play critical roles in maintaining blood glucose levels, providing energy, and regulating metabolic balance. The biosynthesis of unsaturated fatty acids involves the catalytic action of various enzymes. Unsaturated fatty acids are essential components of cell membranes, crucial for maintaining membrane fluidity and stability. Additionally, unsaturated fatty acids participate in various biological processes, including signal transduction, cell adhesion, and inflammatory responses.

Through our examination of single-cell sequencing data, we discovered various cellular subpopulations within osteosarcoma tissue, highlighting the metabolic diversity present among these groups. Notably, the metabolic pathways of GPI-anchor biosynthesis, glycosaminoglycan biosynthesis (heparan sulfate/heparin), glycolysis/gluconeogenesis, and the biosynthesis of unsaturated fatty acids were highly activated in osteoblastic cells. These findings not only enhance our understanding of the metabolic characteristics of osteosarcoma but also provide clues for the development of new therapeutic strategies. Furthermore, we conducted a detailed analysis of the osteoblastic cell subpopulations. Through dimensionality reduction and clustering, we identified six subpopulations, revealing heterogeneity in gene expression among them. Results from CytoTRACE2 and pseudotime analyses indicated differences in differentiation potential and developmental trajectories among the various subpopulations. These findings illuminate the heterogeneity among osteosarcoma cells and lay a foundation for subsequent functional studies and therapeutic target identification.

In our differential expression and enrichment analyses, we discovered several genes and pathways that exhibited significant aberrant expression in osteosarcoma, closely related to the biological processes of malignant proliferation, invasion, and metastasis. For instance, GO analysis results indicated enrichment in the tumor tissue concerning cellular signaling and regulation (small GTPase-mediated signal transduction, nucleoside triphosphatase regulator activity, GTPase regulator activity), cellular structure and movement (extracellular structure organization, external encapsulating structure organization, extracellular matrix organization, cell-substrate junction, focal adhesion, cell leading edge, lamellipodium), skeletal and connective tissue development (bone development, collagen binding), and intracellular structure and function (endoplasmic reticulum lumen, actin binding). In the KEGG analysis, tumor tissues primarily enriched pathways related to the cytoskeleton and structure (cytoskeleton in muscle cells, regulation of actin cytoskeleton, focal adhesion, adherens junction), cell cycle and proliferation (cell cycle), signaling and regulation (Rap1 signaling pathway, Wnt signaling pathway, PI3K-Akt signaling pathway, sphingolipid signaling pathway), and proteoglycans in cancer. These findings are consistent with the biological characteristics of osteosarcoma and provide a basis for subsequent functional research and therapeutic target selection.

Through WGCNA analysis, we identified co-expression modules of genes associated with osteosarcoma metabolism and characterized key genes within these modules. These fundamental genes are essential in regulating the metabolism of osteosarcoma and could act as possible therapeutic targets. GO enrichment analysis of these core genes revealed their enrichment in nuclease activity and DNA metabolism (nuclease activity, ATP-dependent activity acting on DNA, snRNA 3’-end processing), redox and metabolism (oxidoreductase activity, carbon-nitrogen lyase activity), signaling and receptor binding (activin receptor binding), cellular structure and connections (centriole, tight junction, bicellular tight junction, lateral element), intracellular transport and membrane structures (trans-Golgi network membrane, clathrin coat, intraflagellar transport particle, integrator complex, intraflagellar transport particle B), and cell cycle regulation (negative regulation of the cell cycle). This further elucidates the complexity of the pathogenesis of osteosarcoma.

Based on the analysis above, we constructed a risk signature model for osteosarcoma patients using data from the TARGET-OS cohort. This model integrates core genes identified through WGCNA, marker genes obtained from single-cell analyses, and differentially expressed genes. The optimal prognostic gene combinations were selected using Cox regression and LASSO regression analyses. Among the model’s genes, PTK7 serves as a co-receptor in the Wnt signaling pathway, regulating cell polarity, movement, and migration, and is upregulated in various cancers. COL5A2 and COL5A1 encode the α2 and α1 chains of type V collagen, respectively, and are important components of the extracellular matrix, associated with wound healing and tissue regeneration. ALPL encodes alkaline phosphatase, which is involved in bone mineralization. MDFI is related to cytoskeletal remodeling and cell motility. SDC2, a type of chondroitin sulfate proteoglycan, plays a role in cell adhesion and signaling. TMSB4XP8 (thymosin β4) has anti-inflammatory properties and promotes wound healing. SPP1 encodes osteopontin, which is involved in bone metabolism and tumorigenesis. This risk signature model demonstrated strong predictive performance across three independent cohorts (AUC > 0.6), providing significant support for prognosis assessment and personalized treatment in osteosarcoma patients.

To further validate the clinical relevance of this risk signature model, we conducted comprehensive biological and immunological analyses. Our findings indicated that the high-risk group exhibited poorer prognostic characteristics across several metrics, such as a higher number of deceased patients and elevated risk scores among those who died, while the risk scores and patient proportions across different age groups and stages showed no statistically significant differences. Furthermore, the group identified as high-risk demonstrated notably lower scores compared to the low-risk group across various cancer hallmark pathways, such as allograft rejection, apoptosis, complement, IL2-STAT5 signaling, inflammatory response, interferon gamma response, peroxisome pathways, and reactive oxygen species pathways. This further validates the model’s effectiveness.

Moreover, immune cell infiltration levels were assessed using the CIBERSORT algorithm, revealing that the high-risk group exhibited lower levels of immune cell infiltration. The actual ratio of high-risk individuals in the TIDE score was markedly less than that found in the low-risk group, suggesting increased capabilities for tumor immune evasion and variations in immunotherapy responsiveness among the high-risk population. Sensitivity analyses showed that high-risk patients were less responsive to treatments with AZD5582, JAK, Ruxolitinib, and Staurosporine, suggesting that these patients may require personalized therapeutic regimens.

In summary, this study systematically analyzed gene expression in osteosarcoma, elucidating its pathogenic mechanisms and metabolic characteristics, and established a risk signature model for osteosarcoma patients based on the TARGET-OS cohort. This model provides robust support for prognosis assessment and individualized treatment strategies, while also laying the groundwork for further functional studies and therapeutic target identification. Our study provides valuable insights into the heterogeneity of osteosarcoma cell characteristics and metabolic states, with potential clinical implications for targeted therapy and prognostic assessment. The risk signature model developed based on core genes, marker genes, and differentially expressed genes shows robust predictive performance across independent cohorts, suggesting its feasibility in clinical settings. This model could aid in patient stratification, guiding personalized treatment strategies and improving clinical outcomes. Although this research contributes significantly to our understanding of osteosarcoma pathogenesis and points toward new therapeutic strategies, it is not without limitations, such as a limited sample size and a lack of *in vitro* or *in vivo* experimental validation. Future studies will aim to expand the sample size and conduct more extensive experimental validations to corroborate our findings.

## 5 Conclusion

In summary, this study systematically analyzes gene expression in osteosarcoma, elucidating its pathogenesis and metabolic characteristics. We developed a risk signature model for osteosarcoma patients based on the TARGET-OS cohort. This model offers robust support for prognostic assessment and personalized treatment in osteosarcoma patients, laying the groundwork for further functional studies and therapeutic target identification. The results of our research not only deepen our comprehension of the mechanisms underlying the disease but also offer valuable insights and hints for formulating new treatment approaches.

## Data Availability

The original contributions presented in the study are included in the article/**Supplementary Material**. Further inquiries can be directed to the corresponding author.

## References

[1] Yu L Chen Y Yuan S Cao Y Bi Z. Peiminine induces G0/G1-phase arrest, apoptosis, and autophagy via the ROS/JNK signaling pathway in human osteosarcoma cells in vitro and in vivo. Front Pharmacol. (2021) 12:770846. doi: 10.3389/fphar.2021.770846 34867399 PMC8633898

[2] Liao J Shi K Jia Y Wu Y Qian Z. Gold nanorods and nanohydroxyapatite hybrid hydrogel for preventing bone tumor recurrence via postoperative photothermal therapy and bone regeneration promotion. Bioact Mater. (2021) 6:2221–30. doi: 10.1016/j.bioactmat.2021.01.006 PMC7829101 33553811

[3] Zhao J Pan B Zhou X Wu C Hao F Zhang J. Polygonum cuspidatum inhibits the growth of osteosarcoma cells via impeding Akt/ERK/EGFR signaling pathways. Bioengineered. (2022) 13:2992–3006. doi: 10.1080/21655979.2021.2017679 35129428 PMC8974113

[4] Li S Pei Y Wang W Liu F Zheng K Zhang X. Circular RNA 0001785 regulates the pathogenesis of osteosarcoma as a ceRNA by sponging miR-1200 to upregulate HOXB2. Cell Cycle. (2019) 18:1281–91. doi: 10.1080/15384101.2019.1618127 PMC6592237 31116090

[5] Gokulnath P de Cristofaro T Manipur I Di Palma T Soriano AA Guarracino MR. Long non-coding RNA HAND2-AS1 acts as a tumor suppressor in high-grade serous ovarian carcinoma. Int J Mol Sci. (2020) 21. doi: 10.3390/ijms21114059 PMC7312972 32517089

[6] Leng J Song Q Zhao Y Wang Z. miR−15a represses cancer cell migration and invasion under conditions of hypoxia by targeting and downregulating Bcl−2 expression in human osteosarcoma cells. Int J Oncol. (2018) 52:1095–104. doi: 10.3892/ijo.2018.4285 PMC5843390 29484432

[7] Huang Z Chen H Wang S Wei H Wang X Shen R. NLRP3 overexpression associated with poor prognosis and presented as an effective therapeutic target in osteosarcoma. Front Pharmacol. (2021) 12:724923. doi: 10.3389/fphar.2021.724923 34393801 PMC8355743

[8] Ma Y Zhang S Wu Z Sun W. Metabolic variations in brown rice fertilised with different levels of nitrogen. Foods. (2022) 11. doi: 10.3390/foods11213539 PMC9653856 36360153

[9] van Veen M Matas-Rico E van de Wetering K Leyton-Puig D Kedziora KM De Lorenzi V. Sidenius N et al: Negative regulation of urokinase receptor activity by a GPI-specific phospholipase C in breast cancer cells. Elife. (2017) 6. doi: 10.7554/eLife.23649 PMC5576486 28849762

[10] Wolpin BM O'Reilly EM Ko YJ Blaszkowsky LS Rarick M Rocha-Lima CM. Macarulla T et al: Global, multicenter, randomized, phase II trial of gemcitabine and gemcitabine plus AGS-1C4D4 in patients with previously untreated, metastatic pancreatic cancer. Ann Oncol. (2013) 24:1792–801. doi: 10.1093/annonc/mdt066 PMC3716216 23448807

[11] Yu Y Williams A Zhang X Fu L Xia K Xu Y. Specificity and action pattern of heparanase Bp, a β-glucuronidase from Burkholderia pseudomallei. Glycobiology. (2019) 29:572–81. doi: 10.1093/glycob/cwz039 PMC6639543 31143933

[12] Aguilar-Calvo P Sevillano AM Bapat J Soldau K Sandoval DR Altmeppen HC. Sanchez H et al: Shortening heparan sulfate chains prolongs survival and reduces parenchymal plaques in prion disease caused by mobile, ADAM10-cleaved prions. Acta Neuropathol. (2020) 139:527–46. doi: 10.1007/s00401-019-02085-x PMC7036335 31673874

[13] Yeh CL Yang PJ Lee PC Wu JM Chen PD Huang CC. Intravenous glutamine administration improves glucose tolerance and attenuates the inflammatory response in diet-induced obese mice after sleeve gastrectomy. Nutrients. (2020) 12. doi: 10.3390/nu12103192 PMC7603202 33086562

[14] Wang Y Hou Y Qiu J Li Z Zhao J Tong X. A quantitative acetylomic analysis of early seed development in rice (Oryza sativa L.). Int J Mol Sci. (2017) 18. doi: 10.3390/ijms18071376 PMC5535869 28654018

[15] Li S Yue Q Zhou S Yan J Zhang X Ma F. Trehalose contributes to gamma-linolenic acid accumulation in cunninghamella echinulata based on de novo transcriptomic and lipidomic analyses. Front Microbiol. (2018) 9:1296. doi: 10.3389/fmicb.2018.01296 29963034 PMC6013572

[16] Lim JM Vikramathithan J Hwangbo K Ahn JW Park YI Choi DW. Threonine 286 of fatty acid desaturase 7 is essential for ω-3 fatty acid desaturation in the green microalga Chlamydomonas reinhardtii. Front Microbiol. (2015) 6:66. doi: 10.3389/fmicb.2015.00066 25699037 PMC4318421

[17] Coutinho FH Cabello-Yeves PJ Gonzalez-Serrano R Rosselli R López-Pérez M Zemskaya TI. New viral biogeochemical roles revealed through metagenomic analysis of Lake Baikal. Microbiome. (2020) 8:163. doi: 10.1186/s40168-020-00936-4 33213521 PMC7678222

[18] Lei T Qian H Lei P Hu Y. Ferroptosis-related gene signature associates with immunity and predicts prognosis accurately in patients with osteosarcoma. Cancer Sci. (2021) 112:4785–98. doi: 10.1111/cas.v112.11 PMC8586685 34506683

[19] Chen Q Tan Y Zhang C Zhang Z Pan S An W. A weighted gene co-expression network analysis-derived prognostic model for predicting prognosis and immune infiltration in gastric cancer. Front Oncol. (2021) 11:554779. doi: 10.3389/fonc.2021.554779 33718128 PMC7947930

[20] Kim KD Tanizawa H De Leo A Vladimirova O Kossenkov A Lu F. Epigenetic specifications of host chromosome docking sites for latent Epstein-Barr virus. Nat Commun. (2020) 11:877. doi: 10.1038/s41467-019-14152-8 32054837 PMC7018943

[21] Liu W Hao Y Tian X Jiang J Qiu Q. The role of NR4A1 in the pathophysiology of osteosarcoma: A comprehensive bioinformatics analysis of the single-cell RNA sequencing dataset. Front Oncol. (2022) 12:879288. doi: 10.3389/fonc.2022.879288 35965537 PMC9371594

[22] Diaz-Papkovich A Anderson-Trocmé L Gravel S. A review of UMAP in population genetics. J Hum Genet. (2021) 66:85–91. doi: 10.1038/s10038-020-00851-4 33057159 PMC7728596

[23] Qi XT Li YL Zhang YQ Xu T Lu B Fang L. Yang B et al: KLF4 functions as an oncogene in promoting cancer stem cell-like characteristics in osteosarcoma cells. Acta Pharmacol Sin. (2019) 40:546–55. doi: 10.1038/s41401-018-0050-6 PMC6461873 29930276

[24] Wang L Huang X You X Yi T Lu B Liu J. Nanoparticle enhanced combination therapy for stem-like progenitors defined by single-cell transcriptomics in chemotherapy-resistant osteosarcoma. Signal Transduct Target Ther. (2020) 5:196. doi: 10.1038/s41392-020-00248-x 32973147 PMC7518281

[25] Liu D Liu Y Zhang D Chen X Liu Q Xiong B. Fang H et al: quantitative proteome profiling reveals cellobiose-dependent protein processing and export pathways for the lignocellulolytic response in *Neurospora crassa* . Appl Environ Microbiol. (2020) 86. doi: 10.1128/AEM.00653-20 PMC7376555 32471912

[26] Lin Z Xie F Triviño M Zhao T Coppens F Sterck L. Self-incompatibility requires GPI anchor remodeling by the poppy PGAP1 ortholog HLD1. Curr Biol. (2022) 32:1909–1923.e1905. doi: 10.1016/j.cub.2022.02.072 35316654 PMC7612714

[27] Patel VN Pineda DL Berenstein E Hauser BR Choi S Prochazkova M. Kulkarni A et al: Loss of Hs3st3a1 or Hs3st3b1 enzymes alters heparan sulfate to reduce epithelial morphogenesis and adult salivary gland function. Matrix Biol. (2021) 103-104:37–57. doi: 10.1016/j.matbio.2021.10.002 34653670 PMC8629026

[28] Liu ZH Li T He QY Sun Z Jiang Y. Role of mitochondria in regulating lutein and chlorophyll biosynthesis in chlorella pyrenoidosa under heterotrophic conditions. Mar Drugs. (2018) 16. doi: 10.3390/md16100354 PMC6213193 30274203

[29] Chen J Mitra R Zhang S Zuo Z Lin L Zhao D. Unusual phosphoenolpyruvate (PEP) synthetase-like protein crucial to enhancement of polyhydroxyalkanoate accumulation in haloferax mediterranei revealed by dissection of PEP-pyruvate interconversion mechanism. Appl Environ Microbiol. (2019) 85. doi: 10.1128/AEM.00984-19 PMC6752022 31350314

[30] Arends CJ Wilson LH Estrella A Kwon OS Weinstein DA Lee YM. A mouse model of glycogen storage disease type IX-beta: A role for phkb in glycogenolysis. Int J Mol Sci. (2022) 23. doi: 10.3390/ijms23179944 PMC9456097 36077341

